# Impact of acetylcholinesterase inhibitors on the occurrence of acute coronary syndrome in patients with dementia

**DOI:** 10.1038/srep15451

**Published:** 2015-11-18

**Authors:** Ping-Hsun Wu, Yi-Ting Lin, Po-Chao Hsu, Yi-Hsin Yang, Tsung-Hsien Lin, Chia-Tsuan Huang

**Affiliations:** 1 Division of Nephrology, Department of Internal Medicine, Kaohsiung Medical University Hospital, Kaohsiung, Taiwan; 2 Department of Family Medicine, Kaohsiung Medical University Hospital, Kaohsiung, Taiwan; 3 Division of Cardiology, Department of Internal Medicine, Kaohsiung Medical University Hospital, Kaohsiung, Taiwan; 4 Institute of Clinical Medicine, College of Medicine, Kaohsiung Medical University, Kaohsiung, Taiwan; 5 Faculty of Medicine, College of Medicine, Kaohsiung Medical University, Kaohsiung, Taiwan; 6 School of Pharmacy, College of Pharmacy, Kaohsiung Medical University, Kaohsiung, Taiwan

## Abstract

The study aimed to investigate the association of acetylcholinesterase inhibitors (AChEIs) use with the risk of acute coronary syndrome (ACS). We conducted a population-based retrospective cohort study of dementia patients during 1 January 1999 to 31 December 2008 using the National Health Insurance Database in Taiwan. New AChEI users during the study period were matched with AChEI nonusers in age-matched and gender-matched cohorts. The risk of ACS associated with use of AChEIs was analyzed using modified Kaplan-Meier analysis and Cox proportional hazard models after adjustment for competing death risk. Use of AChEIs was associated with a lower incidence of ACS (212.8/10,000 person-years) compared to the matched reference cohort (268.7/10,000 person-years). The adjusted hazard ratio for ACS in patients with dementia treated with AChEIs was 0.836 (95% confidence interval, 0.750–0.933; *P* < 0.001). Further sensitivity analysis of different study populations demonstrated consistent results. A statistical dose–response relationship for AChEI use and ACS risk was significant for the patients with dementia. In patients with dementia, AChEI treatment was associated with decreased risk of ACS.

Dementia, a syndrome of progressive neurodegenerative disorder, is an emerging public health problem because of the increasing number of patients, long duration, high cost, and high mortality rates. The existence of a link between atherosclerosis and dementia has been suggested because these diseases share conventional and genetic risk factors^1^. For example, myocardial infarction and Alzheimer disease (AD) share genetic backgrounds involving abnormalities in cholesterol metabolism and an upregulation in inflammation^2^. It is conceivable that vascular risk factors would be more prevalent in patients with dementia than in the general population and that the incidence of vascular events would increase in dementia.

Acetylcholinesterase inhibitors (AChEIs), which include the drugs donepezil, rivastigmine, and galantamine, act by blocking the enzyme acetylcholinesterase and reducing the breakdown of acetylcholine. AChEIs have been shown to slow the decline of cognitive status and are currently approved for the treatment of AD^3^. AChEIs have also been reported to be effective in other forms of dementia, including vascular dementia^4^. Several previous experimental studies suggested that AChEIs also have anti-inflammatory properties^5^,^6^, protect endothelial cells^7^, and improve cardiac vagal activity^8^,^9^. Because inflammation and endothelial cells play important roles in acute coronary syndrome (ACS), we hypothesizes that the risk of ACS is decreased in dementia with AChEIs used. For this reason, the study aim was to conduct a nationwide cohort study in Taiwan to investigate the association between AChEI use and risk of ACS.

## Methods

### Data source

This population-based cohort study utilized the Taiwan National Health Insurance Research Database (NHIRD), which consists of detailed health care data for more than 23.7 million enrollees, representing more than 99% of the entire population of Taiwan. The database has previously been used for epidemiological research, and information on prescription use, diagnoses, and hospitalizations is of high quality^10^,^11^,^12^. The NHIRD also includes a registry system for “catastrophic illnesses,” including cancer, end-stage renal disease, AD, and several autoimmune diseases. The database contains all relevant information about catastrophic illness status, including diagnostic codes based on the *International Classification of Disease, Ninth Revision* (ICD-9), dates of diagnosis, dates of death, dates of clinic visits, details of prescriptions, expenditure amounts, and outpatient/inpatient claims data. The registry is comprehensive because each individual registered in the database of catastrophic illnesses is exempted from any copayment for treatment. The study was approved by the Institutional Review Board of Kaohsiung Medical University Hospital (KMUH-IRB-EXEMPT-20130062). The methods were carried out in accordance with the approved guidelines.

### Study population and cohort

From the catastrophic illness patient registry, we selected 45,395 patients with dementia diagnosed and were defined as those who underwent catastrophic illness registration for dementia (ICD-9 code 290, 331.0) between 1 January 1999 and 31 December 2008. Individuals younger than 50 years (n = 689) were excluded. Of a total of 44,706 patients with dementia, there were 9070 patients treated with AChEIs and 35,636 patients without treatment. We matched each of these patients with an untreated control selected from the same catastrophic registry according to age, sex, and index date of AChEI prescription.

### Acetylcholinsterase inhibitor use

Dementia patients received prescriptions for AChEIs (N06DA02, N06DA03, and N06DA04 according to the anatomical therapeutic chemical classification system). In Taiwan, patients with claims for AChEI prescriptions must have dementia diagnosed by a neurologist or psychiatrist according to the criteria of ICD-9, the National Institute of Neurological and Communicative Disorders and Stroke–Alzheimer’s Disease and Related Disorders Association, or the Diagnostic and Statistical Manual of Mental Disorder–IV. A patient who applies for drug reimbursement for the first time must have the diagnosing physician complete case studies of the patient’s detailed medical records, biochemistry data (including complete blood cell count, venereal disease laboratory results, blood urea nitrogen, creatinine, alanine aminotransferase, aspartate aminotransferase, thyroxine, and thyrotropin), and neuroimages (at least one report of computed tomography, magnetic resonance imaging, or Hachinski ischemic score). The detailed description of the application and review process for AChEI reimbursement has been reviewed in a previous study^13^.

Exposure to AChEI was quantified in terms of the defined daily dose (DDD). Based on the World Health Organization definition, a DDD is the mean daily maintenance dose of a drug used for its main indication. By using the following formula, we can compare any AChEI based on the same standard: (total amount of drug)/(amount of drug in a DDD) = number of DDDs^14^. The DDD does not necessarily reflect the recommended or prescribed daily dose. Cumulative DDDs (cDDDs), the sum of dispensed DDDs of any AChEI, served as the duration of AChEI exposure to compare the use of the drug to the risk of ACS. To examine the dose–response relationship, we defined three dosage groups in each cohort: less than 28, 28 to 365, and more than 365 cDDDs. Patients who used AChEIs for less than 28 cDDDs were considered AChEI nonusers in the dose–response relationship models.

### Comorbidities and exposure to confounding medications

Baseline demographic data for all individuals in both cohorts were obtained from inpatient and outpatient reimbursement data in NHIRD. We identified the following comorbidities as potential confounders: diabetes mellitus; hypertension; hyperlipidemia; coronary artery disease; heart failure; atrial fibrillation; peripheral artery disease; cerebrovascular disease; chronic obstructive pulmonary disease; chronic kidney disease; malignancy; and depression (Supplemental Table S1). The definition of diabetes mellitus, hypertension, and hyperlipidemia required both the specific ICD-9-CM codes and the use of disease-defining medications for a minimum of 90 days. Socio-demographic characteristics (age, sex, income, and the level of urbanization) were also taken into consideration in our analysis. Urbanization levels in Taiwan are divided into three strata according to the Taiwan National Health Research Institute publications. Economic status was classified into three categories: fixed premium and dependent; less than New Taiwan dollars (NTD) 20,000 monthly; or NTD 20,000 or more monthly (US$1 = NTD32.1 in 2008).

We also retrieved details regarding medications used during the cohort observation period, including antiplatelets, antihypertensive drugs (angiotensin-converting enzyme inhibitors, angiotensin receptor blockers, beta-blockers, thiazides, and calcium channel blockers), statins, nonsteroidal anti-inflammatory drugs (traditional nonsteroidal anti-inflammatory drugs and cyclooxygenase-2 selective inhibitors), antiacid drugs (proton pump inhibitors and histamine-2 receptor antagonists), antidepressants, and antipsychotics.

### Measurement of outcomes

Our primary and secondary outcomes were the occurrence of ACS and all-cause death during the study period. ACS was defined as admission to a hospital for ACS, which has been validated in previous study^15^,^16^. If a patient was hospitalized more than once for ACS, then only the first episode of ACS was used in the analysis. The study end points were followed until primary outcome, death, or 2009.

### Sensitivity analyses

To assess the robustness of our results, we performed a series of sensitivity analyses that included: (1) focusing on individuals without a history of ACS; (2) focusing on individuals without a history of ACS and cerebrovascular disease; and (3) focusing on individuals without a history of major cardiovascular risks, including coronary artery disease, cerebrovascular disease, heart failure, atrial fibrillation, and peripheral artery disease. These sensitivity analyses were conducted with the purpose of examining whether the main findings were robust for different assumptions and study designs.

### Statistical analysis

Baseline descriptive data are described as mean ± standard deviation for continuous variables and as frequency and percentage for categorical variables. All subsequent analyses were performed in the matched samples using methods appropriate for the analysis of matched data for estimating the treatment effect and its statistical significance. Because the recurrence of ACS with AChEI treatment and comparison groups had competing risk for death, we used the modified Kaplan-Meier method^17^ to estimate the cumulative incidence rates of ACS for the two groups. Differences between the two groups were compared by the modified two-tailed log-rank test. After confirming the assumption of proportional hazards by plotting the graph of the survival function versus the survival time and the graph of the log [-log(survival)] versus the log of survival time, we constructed modified Cox proportional hazards models (Fine and Gray competing-risk models^18^) to derive hazard ratios (HRs) and 95% confidence intervals (CIs) in relation to the primary outcomes. We adjusted the models for living area, socioeconomic status, comorbidity, and other medications used during the observation period. All analyses were performed using SAS statistical software (version 9.2; SAS Institute Inc., www.sas.com). Calculations of cumulative incidence in the competing risk analysis were performed using the cmprsk package R (http://cran.r-project.org/web/packages/cmprsk/index.html). All statistical tests were two-sided. *P* < 0.05 was considered statistically significant.

## Results

### Baseline characteristics

A total of 45,395 patients had dementia diagnosed between 1 January 1999 and 31 December 2008. Of these, we excluded 689 patients who were younger than 50 years old. After the age-matching and sex-matching process, 9035 AChEI users and 9035 AChEI nonusers were enrolled. Table 1 shows the baseline characteristics of the study population. Patients who received AChEIs were more likely to live in a city, were more likely to have hypertension, hyperlipidemia, coronary artery disease, peripheral artery disease, malignancy, or depression, and were less likely to have a history of other underlying diseases, including heart failure, atrial fibrillation, and cerebrovascular disease (Table 1). Higher proportion of the patients in the AChEI users group received more concomitant medical treatment than those who did not use AChEIs (Table 1). The mean and median follow up time of AChEI use was 4.6 years and 4.5 years.

### Cumulative incidence and risk of ACS occurrence during follow-up

**Figure 1:**
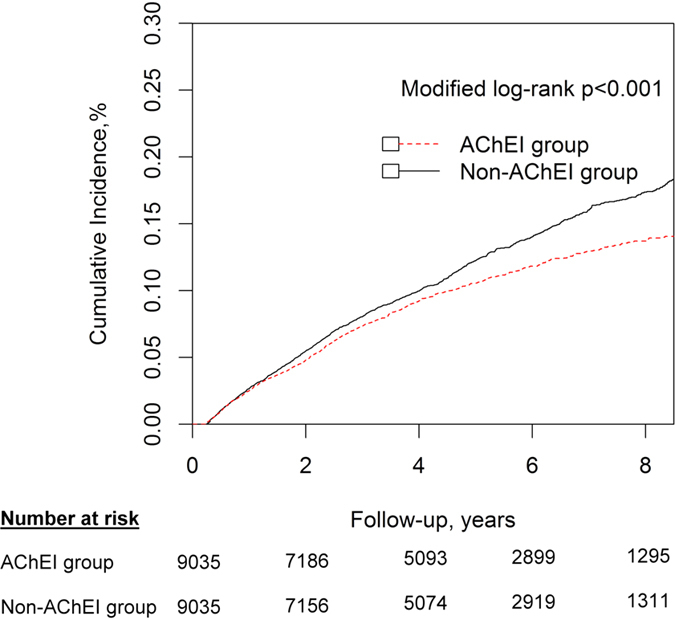
Cumulative incidences of acute coronary syndrome (ACS) for dementia patients who were treated with or without acetylcholinesterase inhibitors. Data were compiled after adjustment for competing mortality. For cumulative incidences of ACS, calculation and comparison of competing risk data ratios were conducted using modified Kaplan-Meier and Gray methods.

During the 10-year follow-up period, ACS was diagnosed in 890 AChEI users (9.85%), which corresponded to an incidence rate of 212.8 (95% CI, 199.1–227.1) per 10,000 person-years. Of AChEI nonusers, 1124 patients had ACS (12.44%), which corresponded to an incidence rate of 268.7 (95% CI, 253.3–284.8) per 10,000 person-years (Supplemental Table S2). Using Kaplan–Meier estimates with adjustment for competing risk of mortality, the cumulative incidence rates of ACS were significantly lower for the AChEI users than for the nonusers (modified log-rank *P* < 0.001) (Fig. 1).

Death before the occurrence of ACS was defined as competing mortality. Multivariable analysis by Cox proportional hazards model was further adjusted for competing mortality. In the multivariable Cox regression analysis adjusting for urbanization, and socioeconomic status (model 1), AChEI users had a lower risk of ACS (adjusted HR, 0.815; 95% CI, 0.746–0.891; *P* < 0.001) than nonusers. Compared to AChEI nonusers, the association of a lower risk of ACS with AChEI use remained significant even after additionally adjusting for comorbid conditions (including diabetes mellitus, hypertension, hyperlipidemia, coronary artery disease, heart failure, atrial fibrillation, peripheral artery disease, cerebrovascular disease, chronic obstructive pulmonary disease, chronic kidney disease, malignancy, and depression; adjusted HR, 0.752; 95% CI, 0.686–0.823; *P* < 0.001; model 2) and medications, including antiplatelets, antihypertensive drugs, statins, nonsteroidal anti-inflammatory drugs, antiacid drugs, antidepressants, and antipsychotics (adjusted HR, 0.836; 95% CI, 0.750–0.933; *P* < 0.001; model 3) (Table 2). For the adjusted HR, AChEI users had a lower risk of all-cause mortality compared with nonusers after adjusting for urbanization, and socioeconomic status (adjusted HR, 0.905; 95% CI, 0.849–0.964; *P* = 0.002; model 1) and after further adjusting for comorbidity (adjusted HR, 0.891; 95% CI, 0.836–0.951; *P* < 0.001; model 2), but HR was nonsignificant after further adjusting for concomitant medications (adjusted HR, 0.957; 95% CI, 0.884–1.036; *P* = 0.270; model 3) (Table 2).

A dose–response relationship between AChEI use and ACS risk was found in patients with dementia when they were divided into three groups according to yearly AChEI cDDD. The adjusted HRs were 0.843 (95% CI, 0.742–0.957; *P* = 0.009) and 0.856 (95% CI, 0.753–0.973; *P* = 0.017) for patients with 28 to 365 cDDDs and for patients with more than 365 cDDDs compared with those with less than 28 cDDDs. There was a trend toward ACS risk reduction with increasing cDDDs of AChEIs (*P* < 0.001) (Table 3).

### Subgroup analyses

**Figure 2:**
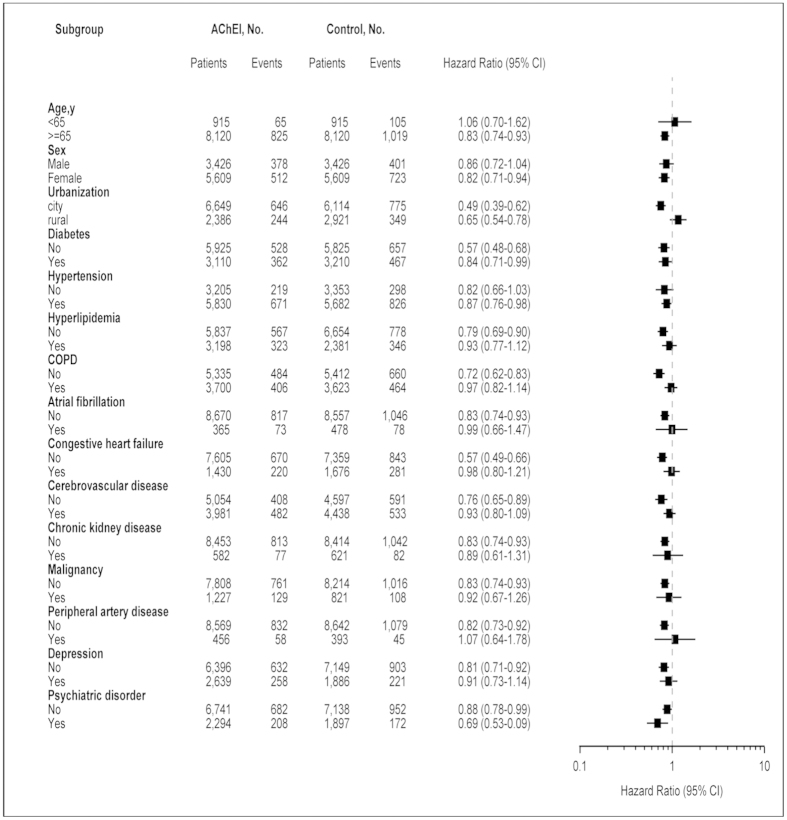
Stratified analysis for acute coronary syndrome (ACS). The risk of ACS in dementia patients with and without acetylcholinesterase inhibitors use (presented as hazard ratios and 95% confidence intervals) is shown, stratified by the baseline characteristics.

Figure 2 shows the fully adjusted HR in relation to the risk of ACS for AChEI users compared with nonusers in the various subgroup analyses. AChEI users had a lower risk of ACS in nearly all subgroups except for patients younger than age 65 years, males, rural live area, and those with hyperlipidemia, chronic obstructive pulmonary disease, atrial fibrillation, chronic kidney disease, peripheral artery disease, and depression.

### Sensitivity analyses

We tested the robustness of our study with a series of sensitivity analyses. The sensitivity analysis that excluded patients with history of ACS found the risk of ACS to be lower among patients who used AChEIs than among nonusers. The risk remained decreased in the sensitivity analysis that excluded ACS and cerebrovascular history or major cardiovascular risks (including coronary artery disease, cerebrovascular disease, heart failure, atrial fibrillation, peripheral artery disease) (Supplemental Table S3).

## Discussion

The present study found significantly decreased risk of ACS associated with use of AChEIs in patients with dementia. There was a statistically significant inverse trend between the dose and the cumulative incidences and HRs of ACS. Further sensitivity analysis of different subpopulations showed that the results were consistent. Despite stratification and adjustment for several risk factors, the evidence derived from a retrospective cohort study is generally lower in statistical quality than that from randomized trials because the nonrandomized design leaves a risk for residual confounding factors.

This present study revealed that AChEI treatment could protect dementia patients from ACS. Several potential mechanisms have been investigated. First, endothelial function is impaired in patients with AD^19^ and AChEIs may have an adjunctive protective role in endothelial dysfunction^20^ based on antiapoptotic effects on endothelial cells and mechanisms against oxidative stress–induced cytotoxicity^7^. Second, atherosclerosis is an inflammatory disease^21^, so the anti-inflammatory effect of AChEIs attributable to reduced acetylcholine breakdown is of interest^22^,^23^. AChEIs attenuate systemic inflammatory responses through inhibiting the production of pro-inflammatory cytokines from activated macrophages and other immune cells^24^. Thus, AChEIs are associated with reduction in the serum cytokine level^5^,^6^ and may reduce ACS events, as observed in our study. Third, Megakaryocytic cells have been shown to contain components of a nonneuronal cholinergic system, including acetylcholine and acetylcholine esterase. Further study also demonstrated the activation of nicotinic acetylcholine receptors contributes to maintaining intracellular Ca2^+^ levels and supporting platelet activation^25^. Beyond cholinergic anti-inflammation pathway effect by acetylcholinesterase activity, the cholinergic system was also associated with platelet pathway^25^,^26^,^27^. Fourth, an impaired baroreceptor reflex function is associated with mortality and major cardiovascular events in patients after myocardial infarction^28^. Vagal stimulation had an anti-arrhythmia effect^29^, could protect cardiomyocytes from acute hypoxia and ischemia^30^, and improved cardiac function and survival^31^ after myocardial infarction. The enhanced heart acetylcholine availability might counterbalance the diminished cardiac vagal activity^32^. The non-neuronal cardiac cholinergic system may play a protective role in both myocardial cells and the entire heart^33^. The administration of AChEIs reproduced the effects of the vagal nerve stimulation, such as preventing pump failure and cardiac remodeling in animal studies^8^,^9^,^34^. AChEIs also have shown protection against heart failure in clinical studies^35^.

The incidence of ischemic heart disease in clinical trials of AChEIs was not significantly greater than in an equivalent comparison population in randomized controlled studies^36^, even in patients with pre-existing cardiovascular disease^37^. Because myocardial infarctions are rarely reported and there are relatively short follow-up periods in clinical trials, pooling of safety data from these studies could be insufficient to evaluate the cardiovascular-protective effect of AChEIs. Such an effort could be of great value given the results of the present observational study. Our study has several strengths. The use of the NHIRD and data regarding universal prescription drug coverage in patients with dementia provided us with a large representative sample of patients who used AChEIs. The ascertainment of ACS hospitalization and medical comorbidities are complete, objective, and reliable because the NHIRD is a compulsory and universal health care system with a very high coverage rate in Taiwan. In the present study, linking patient data with administrative databases allowed for the follow-up of each patient regardless of further participation, allowing us to avoid attrition over time and minimizing the possibility of recall bias.

A recent observational study in Sweden found that AChEI use was associated with 38% lower risk of myocardial infarction in patients with AD and 36% decreased risk of death^38^. Another hospital based study also showed the survival benefit in donepezil-treated patients^39^. The results were similar to those of the present study, with 18% to 25% lower risk of ACS, but the association between death and use of AChEIs was not significant in the present study. We enrolled Asian patients with a different ethnic background from Europeans for the further longer follow-up period. Even if such a difference existed, AChEIs was associated with lower ACS events. However, several differences in patient characteristics between these two studies should be noted. First, our study enrolled those with higher cardiovascular comorbidities compared to the Swedish study, which excluded patients at high risk for cardiovascular disease^38^. Although use of AChEIs might lower mortality risk, studies addressing the relationship between AChEI use and mortality are conflicting^40^,^41^. The difficulty lies in establishing a cause–effect relationship given the variability in the causes of mortality in advanced age. Second, study population, baseline characteristics, and early or late start of AChEI therapy were different in the two studies. The percentage of our patients with dementia receiving AChEIs was much lower than that of patients in Europe^42^. Also, initiating AChEIs for some patients with AD in Taiwan was delayed, which might have reduced the effect on lowering mortality. Third, the mortality rates of patients with dementia in Taiwan, which population-based studies report as ranging from 32% to 48%^43^,^44^, are higher than those reported for Western populations^45^. The different mortality rates in different countries and races might partially explain the results of AChEI effects on death risk in the present study.

Sensitivity analysis was applied to test the different impact on a variable population. We excluded dementia patients with history of ACS in this present cohort to test the primary prevention effect of AChEI. Furthermore, dementia patients with ACS and history of cerebrovascular disease were excluded to keep a group of patients with lower risk of cardiovascular disease. Finally, dementia patients without any major cardiovascular risks who were at lowest cardiovascular risk were evaluated. The results were consistent in all groups using sensitivity analysis, which means that AChEIs have protective effects against ACS in dementia patients, regardless of cardiovascular risk. The HR tended to be lower in the group with high cardiovascular risk.

Several limitations should be considered. First, the study was a retrospective review of the medical records in a database and was not based on formal cognitive function testing. In addition, AD and vascular dementia often coexist as a mixed dementia, thereby complicating diagnosis. The Alzheimer type or other types of dementia, such as vascular dementia, cannot be exactly and accurately differentiated from dementia in the NHIRD because there is no information available from the diagnostic codes of ICD-9-CM.Dementia coding has been reported to have low sensitivity but high specificity^46^, so we enrolled all dementia patients as our study population. Ascertainment of dementia based on ICD-9 coding was accurate in the present study because it was well-validated in the catastrophic illness registry. However, dementia severity was not available in our study. Second, our study was observational, and the associations found were no proof of a cause–effect result from AChEI use. Although our study was observational, we were able to show an effect after adjusting for a large number of cardiovascular risk factors. Despite our meticulous study design and control measures for confounding factors, bias resulting from unknown confounders may have affected the results. However, the study reflected real-world clinical practice. Third, the diagnosis of various comorbidities was based on claims data and ICD-9 codes, which may be associated with potential misclassification bias. However, the Taiwan Bureau of National Health Insurance performs regular audits of the quality of data captured and of all medical charges, and it imposes heavy penalties for outlier charges or malpractice. Consequently, this ensures data quality and, given the large sample size, the limitation can be dismissed. Fourth, the NHIRD lacks data regarding individual behaviors and information. Thus, other potential confounding factors such as lifestyle, nutrition status, body mass index, medication compliance, laboratory parameters, smoking habits, alcohol use, genetic factors (for example, apolipoprotein E4 genotype), and family history, all of which may contribute to the risk of ACS. Finally, the study included Taiwanese patients only. Whether the findings are also applicable to other ethnic population requires further evaluation. Because of the inclusion of all dementia patients, its external validity for the population without dementia is unknown.

## Conclusion

This observational matched cohort study indicates that use of AChEIs in dementia patients is associated with a decreased risk of an ACS event. Further randomized prospective studies are needed to confirm our findings.

## Additional Information

**How to cite this article**: Wu, P.-H. *et al*. Impact of acetylcholinesterase inhibitors on the occurrence of acute coronary syndrome in patients with dementia. *Sci. Rep*. **5**, 15451; doi: 10.1038/srep15451 (2015).

## Supplementary Material

Supplementary Information

## Figures and Tables

**Table 1:** Baseline characteristics of age- and sex-matched dementia patients using or not using acetylcholinesterase inhibitors.

Characteristic	Age-Matched and Sex-Matched				
	Patients Using AChEIs (n = 9035)		Patients Not Using AChEIs (n = 9035)		*P*
	N	%	N	%	
Age, years					1
50–59	464	5.1	464	5.1	
60–69	1803	20	1803	20	
70–79	4491	49.7	4491	49.7	
≥80	2277	25.2	2277	25.2	
Sex					1
Male	3426	37.9	3426	37.9	
Female	5609	62.1	5609	62.1	
Urbanization level					<0.001
City	6649	73.6	6114	67.7	
Rural	2386	26.4	2921	32.3	
Socioeconomic status					0.941
Low	4939	54.7	4881	54	
Moderate	1942	21.5	2086	23.1	
High	2154	23.8	2068	22.9	
Comorbidities					
Diabetes mellitus	3110	34.4	3210	35.5	0.119
Hypertension	5830	64.5	5682	62.9	0.022
Hyperlipidemia	3198	35.4	2381	26.4	<0.001
Coronary artery disease	1227	13.6	821	9.1	<0.001
Heart failure	1430	15.8	1676	18.6	<0.001
Atrial fibrillation	365	4	478	5.3	<0.001
Peripheral artery disease	466	5.2	393	4.3	0.011
Cerebrovascular disease	3981	44.1	4438	49.1	<0.001
COPD	3700	41	3623	40.1	0.243
Chronic kidney disease	582	6.4	621	6.9	0.245
Malignancy	1227	13.6	821	9.1	<0.001
Depression	2639	29.2	1886	20.9	<0.001
Medication Prescription					
Antiplatelets	2546	28.18	1862	20.61	<0.001
Antihypertensive drugs	4699	52.01	3661	40.52	<0.001
Statin	896	9.92	497	5.5	<0.001
NSAIDs	2782	30.79	1996	22.09	<0.001
Antacid drugs	549	6.08	428	4.74	<0.001
Antidepressants	1965	21.75	921	10.19	<0.001
Antipsychotics	5367	59.4	1119	12.4	<0.001
AChEIs, cDDD					
<28	488	5.4			
28–365	4224	46.8			
≥365	4323	47.8			

AChEIs, acetylcholinesterase inhibitors; cDDD, cumulative defined daily dose; COPD, chronic obstructive pulmonary disease; NSAIDs, nonsteroidal anti-inflammatory drugs.

**Table 2:** Adjusted hazard ratio for acute coronary syndrome among acetylcholinesterase inhibitors users and nonusers in various analytical models.

	Age-Matched and Sex-Matched Cohort		
	Adjusted HR	95% CI	*P*
Acute coronary syndrome*			
Model 1	0.815	0.746–0.891	<0.001
Model 2	0.752	0.686-0.823	<0.001
Model 3	0.836	0.750-0.933	<0.001
Death			
Model 1	0.905	0.849–0.964	0.002
Model 2	0.891	0.836-0.951	<0.001
Model 3	0.957	0.884-1.036	0.270

CI, confidence interval; HR, hazard ratio.

Model 1: adjustment for urbanization level, and socioeconomic status.

Model 2: adjustment for model 1 and comorbidities.

Model 3: adjustment for model 2 and medications in observation period.

^*^Adjusted for competing death risk.

**Table 3:** Incidence rate and crude and adjusted hazard ratios of acute coronary syndrome associated with acetylcholinesterase inhibitor use during the follow-up period in the age-matched and sex-matched dementia cohort.

	No. of Patients With ACS	Incidence Rate (95% CI)*	Crude		Adjusted*		*P* for Trend
			HR (95% CI)	*P*	HR (95% CI)	*P*	
Total duration of AChEI use							<0.001
Nonuser (<28 cDDDs)	1164	265.7(250.7–281.3)	Reference		Reference		
User (28–365 cDDDs)	421	233.7(212.2–256.9)	0.802(0.717–0.897)	0.002	0.843 (0.742-0.957)	0.009	
User (>365 cDDDs)	429	196.5(178.5–215.7)	0.837(0.749–0.935)	<0.001	0.856 (0.753-0.973)	0.017	

AChEI, acetylcholinesterase inhibitor; cDDD, cumulative defined daily dose; CI, confidence interval; HR, hazard ratio.

Incidence rate per 10,000 person-years.

^*^Adjusted for age, sex, urbanization level, socioeconomic status, comorbidities, medications, and competing death risk.
