# Supplementary material for: Impact of acetylcholinesterase inhibitors on the occurrence of acute coronary syndrome in patients with dementia
Source: Sci Rep. 2015 Nov 18;5:15451. doi: 10.1038/srep15451 (PMC4649673; doi:10.1038/srep15451)
Supplement: Supplementary Information [file srep15451-s1.pdf]

Manuscript Title: Impact of acetylcholinesterase inhibitors on the occurrence of acute coronary syndrome in patients with dementia

Authors, Institution, and Affiliations:

Ping-Hsun Wu, MD[1,4], Yi-Ting Lin, MD[2,4], Po-Chao Hsu, MD[3,5], Yi-Hsin Yang, PhD[6], Tsung-Hsien Lin, MD, PhD[3,5,*], Chia-Tsuan Huang, MD[2,*]

[1]Division of Nephrology, Department of Internal Medicine, Kaohsiung Medical University Hospital, Kaohsiung, Taiwan

[2]Department of Family Medicine, Kaohsiung Medical University Hospital, Kaohsiung, Taiwan

[3]Division of Cardiology, Department of Internal Medicine, Kaohsiung Medical University Hospital, Kaohsiung, Taiwan

[4]Institute of Clinical Medicine, College of Medicine, Kaohsiung Medical University, Kaohsiung, Taiwan

[5]Faculty of Medicine, College of Medicine, Kaohsiung Medical University, Kaohsiung, Taiwan

[6]School of Pharmacy, College of Pharmacy, Kaohsiung Medical University, Kaohsiung, Taiwan

[*]**Corresponding authors:**

Tsung-Hsien Lin

Division of Cardiology, Department of Internal Medicine, Kaohsiung Medical

University Hospital

No. 100, Tzyou 1st Road Kaohsiung 807, Taiwan

Telephone number: +886-7-3121101 ext. 7741

Fax number: +886-7-3234845

E-mail address: lth@kmu.edu.tw

and

Chia-Tsuan Huang

Department of Family Medicine, Kaohsiung Medical University Hospital

No. 100, Tzyou 1st Road Kaohsiung 807, Taiwan

Telephone number: +886-7-3121101 ext. 7021

Fax number: +886-7-3116154

E-mail address: chtshu@cc.kmu.edu.tw

Supplemental Table S1. ICD-9 codes used to identify clinical conditions

Diagnosis	Corresponding ICD-9 codes
Dementia	【290】【331.0】
Acute coronary syndrome	【410】【411】
Diabetes mellitus	【250】
Hypertension	【401】–【405】
Hyperlipidemia	【272】
Coronary artery disease	【410】–【414】【429.2】
Heart failure	【398.91】【402.01】【402.11】【402.91】【404.01】【404.03】【404.11】【404.13】【404.91】【404.93】【428】
Atrial fibrillation	【427.3】
Peripheral artery disease	【440.2】–【440.4】【443.9】
Cerebrovascular disease	【430】–【438】
Chronic obstructive pulmonary disease	【491】【492】【496】
Chronic kidney disease	【250.4】【274.1】【403.1】【404.2】【404.3】【580】【582】【583】【585】【586】【587】
Malignancy	【140】–【208】
Depression	【296.2】【296.3】【296.82】【300.4】

	【309.0】【309.1】【311】

ICD, International Classification of Disease.

Supplemental Table S2. Follow-up duration, numbers, and incidence rate of acute coronary syndrome among patients using and not using acetylcholinesterase inhibitors in an age-matched and sex-matched cohort

	Age-Matched and Sex-Matched	
	Patients Using AChEIs (n = 9035)	Patients Not Using AChEIs (n = 9035)
Total follow-up, person-years	41,829.53	41,832.27
Number of acute coronary syndrome	890	1124
Incidence rate per 10,000 person-years (95% CI)	212.8 (199.1–227.1)	268.7 (253.3–284.8)

AChEIs, acetylcholinesterase inhibitors; CI, confidence interval.

Supplemental Table S3. Sensitivity analysis of adjusted HRs of acetylcholinesterase inhibitor use in risk reduction of acute coronary syndrome during the follow-up period in the dementia cohort

	Patients Using AChEIs		Patients Not Using AChEIs			
	No. of ACS	Total No. of Patients	No. of ACS	Total No. of Patients	Adjusted HR (95% CI)*	*P*
Main analysis	890	9035	1124	9035	0.680 (0.620-0.745)	<0.001
Main analysis excluding acute coronary syndrome history	653	8102	848	7697	0.652 (0.586–0.725)	<0.001
Main analysis excluding acute coronary syndrome and cerebrovascular history	332	4720	507	4297	0.638 (0.550–0.739)	<0.001
Main analysis excluding major cardiovascular risks§	561	6689	740	6663	0.677 (0.603–0.760)	<0.001

AChEI, acetylcholinesterase inhibitor; ACS, acute coronary syndrome; CI, confidence interval; HR, hazard ratio.

*Adjusted for age, sex, urbanization level, socioeconomic status, comorbidities, medications, and competing death risk

§Major cardiovascular risks defined as coronary artery disease, cerebrovascular disease, heart failure, atrial fibrillation, peripheral artery disease.
